# Rosarugosides A and D from *Rosa rugosa* Flower Buds: Their Potential Anti-Skin-Aging Effects in TNF-α-Induced Human Dermal Fibroblasts

**DOI:** 10.3390/plants13091266

**Published:** 2024-05-02

**Authors:** Kang Sub Kim, So-Ri Son, Yea Jung Choi, Yejin Kim, Si-Young Ahn, Dae Sik Jang, Sullim Lee

**Affiliations:** 1 College of Korean Medicine, Gachon University, Seongnam 13120, Republic of Korea; kangsub@gachon.ac.kr (K.S.K.); domdada22@gachon.ac.kr (Y.J.C.); 2 Department of Biomedical and Pharmaceutical Sciences, Graduate School, Kyung Hee University, Seoul 02447, Republic of Korea; allosori@khu.ac.kr (S.-R.S.); yezeen@khu.ac.kr (Y.K.); 3 Department of Life Science, College of Bio-Nano Technology, Gachon University, Seongnam 13120, Republic of Korea; sy990303@gachon.ac.kr

**Keywords:** *Rosa rugosa*, depsides, TNF-α, ROS, MMP-1, procollagen type Ι α1

## Abstract

This present study investigated the anti-skin-aging properties of *Rosa rugosa*. Initially, phenolic compounds were isolated from a hot water extract of *Rosa rugosa*’s flower buds. Through repeated chromatography (column chromatography, MPLC, and prep HPLC), we identified nine phenolic compounds (**1**–**9**), including a previously undescribed depside, rosarugoside D (**1**). The chemical structure of 1 was elucidated via NMR, HR-MS, UV, and hydrolysis. Next, in order to identify bioactive compounds that are effective against TNF-α-induced NHDF cells, we measured intracellular ROS production in samples treated with each of the isolated compounds (**1**–**9**). All isolates reduced the level of ROS at a concentration of 10 μM. Particularly, two depsides—rosarugosides A and D (**2** and **1**)—significantly inhibited ROS expression in TNF-α-induced NHDFs compared to the other phenolic compounds. Subsequently, the production of MMP-1 and procollagen type Ι α1 by these two depsides was examined. Remarkably, rosarugoside A (**2**) significantly decreased MMP-1 secretion at all concentrations. In contrast, rosarugoside D (**1**) regulated the expression of procollagen type Ι α1. These findings collectively suggest that *Rosa rugosa* extracts and their isolated compounds, rosarugosides A (**2**) and D (**1**), hold significant potential for protecting against aging and skin damage. Overall, these findings suggest that *Rosa rugosa* extracts and their isolated compounds, rosarugosides A (**2**) and D (**1**), have the potential to prevent and protect against aging and skin damage, although more specific quantitative analysis is needed.

## 1. Introduction

*Rosa rugosa* (family Rosaceae), commonly known as rugosa rose, is native to East Asia and is now widely cultivated worldwide [1]. It is well known for its aromatic flowers and is often used to manufacture rose hip oil, which contains sweet, fragrant, and bioactive volatile oils [2]. It also is commonly used to make teas, wines, and jams, highlighting its many applications in food [3]. In addition to its culinary applications, the flower buds of *R*. *rugosa* have long been used in traditional oriental medicine, particularly for their roles in the expansion of blood vessels and enhancement of microcirculation. These functions have prompted recent scientific interest in the pharmacological activities and bioactive compounds of *R*. *rugosa* [4]. Previous phytochemical studies on *R*. *rugosa* have demonstrated that its flowers are highly abundant in phenolic compounds, such as flavonoids, tannins, and anthocyanins, which are recognized for their antioxidant properties [5]. Among its unique phytochemicals, which is to say rugosin A–G, a series of hydrolyzable tannins characterized by valoneoyl groups have been isolated from the flower petals of *R*. *rugosa*. These have revealed their potential anti-inflammatory, antioxidant, anti-cancer, and antimicrobial properties [6,7,8,9,10]. Additionally, we previously reported on unreported depsides with 2-*O*-(3,4-dihydroxybenzoyl)-2,4,6-trihydroxyphenylacetic acid skeletons from *R*. *rugosa*, which were shown to improve schizophrenia-like symptoms, suggesting the pharmacological utility of *R*. *rugosa* [11]. Yu et al. reported that an extract from *Rosa rugosa* had antioxidant and anti-inflammatory effects, displayed collagenase activity, and inhibited melanin formation in B16F10 mouse melanoma cells [12]. Also, it was reported to have anti-aging, whitening, and moisturizing properties [13,14,15,16]. In this study, we focused on *R. rugosa* extracts as they were expected to be effective in assisting the anti-aging process of the skin.

Aging, a natural biological process influenced by both intrinsic and extrinsic factors, encompasses a decline in cellular and tissue function, alterations in gene expression, and metabolic changes [17]. These aging phenomena include the functional decline of cells and tissues, changes in gene expression, and changes in metabolic processes. Reactive oxygen species (ROS) play a crucial role in intracellular signaling and homeostasis at low levels, maintaining balance inside cells at low concentrations [18,19,20]. However, excessive ROS production, caused by external factors, can lead to oxidative stress, which can accelerate aging and contribute to the occurrence of related diseases [21,22]. In particular, oxidative stress also affects the health and beauty of the skin and is associated with wrinkles and the loss of elasticity [23].

Another important factor associated with wrinkles and the loss of elasticity in the skin is proteolytic enzymes, called matrix metalloproteinases (MMPs) [24]. Among the various types of MMPs, MMP-1 promotes collagen breakdown, contributing to fine wrinkles and the loss of elasticity in the skin [25,26,27].

Collagen is found in a variety of connective tissues, including the skin, cartilage, and tendons, where it plays an important role, supporting the structure and maintaining elasticity [28,29]. The increased breakdown of collagen due to aging and the effects of the external environment can reduce the capacity of the skin and lead to the formation of wrinkles [30]. The interaction between ROS, MMP-1, and collagen forms a core mechanism of skin aging, and regulating this process is important for maintaining skin health.

Previous research has reported that the ethanol extract of *R*. *rugosa* flowers has antioxidant characteristics owing to its bioactive phenolic components, as demonstrated by the DPPH radical scavenging assay and the inhibitory activity of tyrosinase [31]. In our preliminary investigation, hot water extracts of *R*. *rugosa* flower buds suppressed ROS production in tumor necrosis factor-alpha (TNF-α)-stimulated normal human dermal fibroblasts (NHDFs), suggesting potential anti-aging properties. Therefore, this study aimed to validate the anti-aging properties of hot water extracts of *R*. *rugosa* flowers and to identify the bioactive compounds present in the extract.

## 2. Results

### 2.1. Identification of Compounds ***1**–**9*** Isolated from R. rugosa Flower Buds

**Figure 1:**
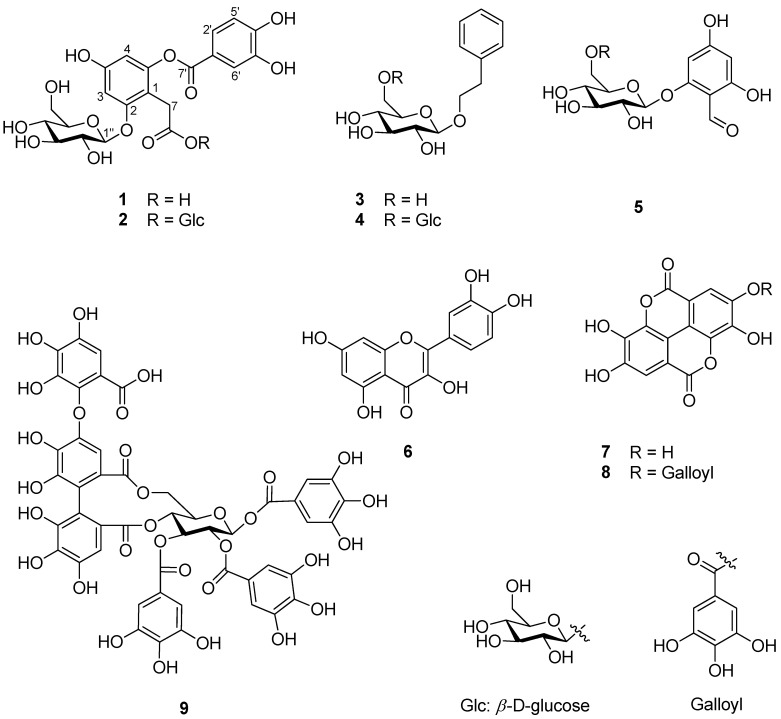
Phenolic compounds **1**–**9** isolated from hot water extracts of *R*. *rugosa* flower buds.

In order to identify the active compounds that were present in hot water extracts of *R*. *rugosa* flower buds, nine compounds were isolated using repetitive chromatography. Via spectroscopic data analysis, compounds **1**–**9** were identified as being phenolic compounds, including one unreported depside (**1**, Figure 1). The known compounds **2**–**9** were determined to be rosarugoside A (**2**) [11], 2-phenylethyl 6-*O*-*β*-d-glucopyranosyl-*β*-d-glucopyranoside (**3**) [32], 2-phenylethyl-*O*-*β*-d-glucopyranoside (**4**) [33], 2-*O*-*β*-d-glucopyranosyl-4,6-dihydroxybenzaldehyde (**5**) [34], quercetin (**6**) [35], ellagic acid (**7**) [36], valoneic acid dilactone (**8**) [37], and rugosin A (**9**) [9] based on comparisons with previously reported data.

**Figure 2:**
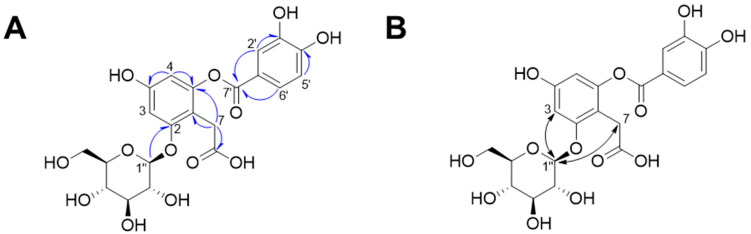
Key HMBC (**A**, 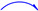) and NOESY (**B**, 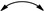) correlations of compound **1**.

Compound **1** was isolated as a dark red powder, and its molecular formula was determined to be C_21_H_22_O_13_ based on its HR-MS-positive ion peak at *m*/*z* 481.0983 (calculated for C_21_H_22_NaO_13_, Figure S1). The UV spectrum of compound **1** revealed that the absorbance maxima were 210, 224, and 271 nm. The ^1^H-NMR spectrum of compound **1** exhibited characteristic resonances for depside: one 1,2,4,6-tetrasubstituted aromatic ring [*δ*_H_ 6.48 (1H, d, *J* = 2.0 Hz, H-5) and 6.67 (1H, d, *J* = 2.5 Hz, H-3)], one 1,3,4-trisubstituted aromatic ring [*δ*_H_ 6.94 (1H, d, *J* = 8.5 Hz, H-5′), 7.55 (1H, d, *J* = 2.5 Hz, H-2′), and 7.59 (1H, dd, *J* = 8.0, 2.0 Hz, H-6′)], and one methylene group [*δ*_H_ 3.61 (2H, m, H-7)]. The presence of an anomeric proton [*δ*_H_ 5.04 (1H, d, *J* = 7.0 Hz, Glc-1″)] and oxygenated proton between *δ*_H_ 3.48 and 3.92 suggested that there was one *β*-configuration sugar in compound **1** (Figure S2). Accordingly, the ^13^C-NMR spectrum revealed 21 carbon signals, including those from a 2-*O*-(3,4-dihydroxybenzoyl)-2,4,6-trihydroxyphenylacetic acid moiety [*δ*_C_ 29.0 (C-7), 101.5 (C-3), 104.6 (C-5), 109.4 (C-1), 115-7 (C-5′), 117.3 (C-2′), 119.9 (C-1′), 124.4 (C-6′), 143.9 (C-3′), 150.2 (C-6), 150.5 (C-4′), 156.4 (C-4), 156.4 (C-2), 166.4 (C-7′), and 176.0 (C-8)] and a *β*-glucopyranosyl group [*δ*_C_ 60.5 (Glc-6″), 69.3 (Glc-4″), 72.8 (Glc-2″), 75.6 (Glc-3″), 76.2 (Glc-5″), 100.9 (Glc-1″)] (Figures S3 and S4). The HMBC and NOESY experiments led to the determination of the substituted position for the *O*-*β*-glucopyranose unit (Figures S5–S8). Specifically, the HMBC experiment revealed a correlation signal between Glc-1″ and C-2 (Figure 2). The NOESY experiment showed correlations between Glc-1″ and H-3 and H-7, confirming the substitution of the *O*-*β*-glucopyranose unit at the C-2 position. Sugar analysis further determined that the absolute configuration of sugar was d-glucose (Figure S9). Therefore, we elucidated that compound **1** was 2-*O*-(3,4-dihydroxybenzoyl)-2,4,6-trihydroxyphenylacetic acid 2-*O*-β-d-glucopyranoside, which is named rosarugoside D.

### 2.2. The Effects of Hot Water Extracts of R. rugosa Flower Buds and Compounds ***1**–**9*** on NHDF Cell Viability 

**Figure 3:**
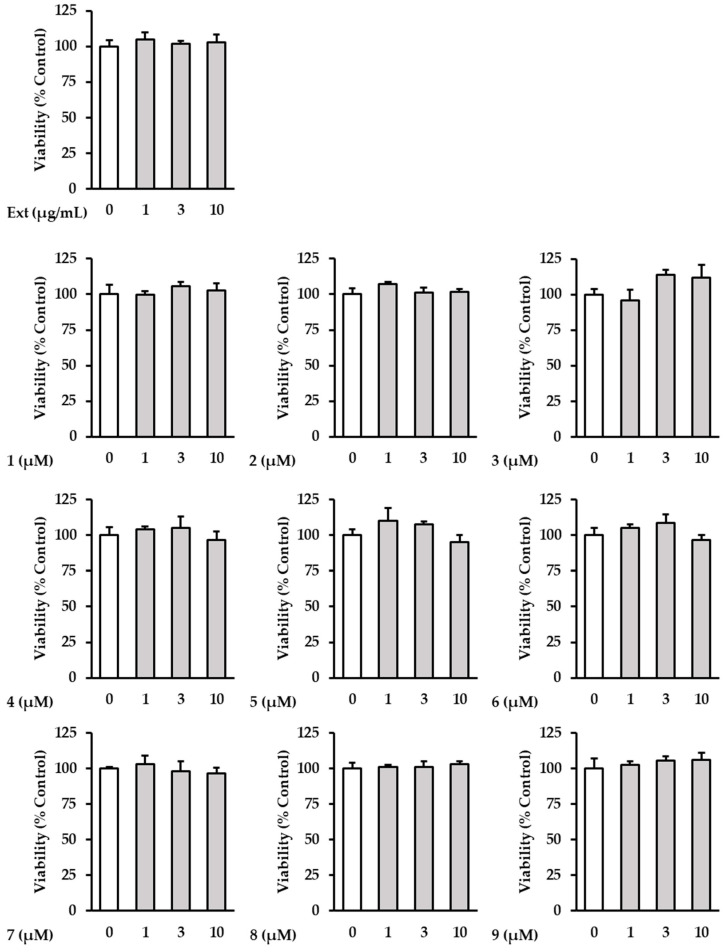
The effects of hot water extracts of *R. rugosa* flower buds (Ext.) and compounds **1**–**9** on NHDF cell viability. The cells were then treated with (1–10 µM) concentrations of the compound for 24 h. The effects of the compounds on cell viability were evaluated using an EZ-Cytox solution. The data are presented as the mean ± SD (*n* = 3).

The cytotoxicity of the samples was further investigated in order to determine the effects of the extract and isolated compounds **1**–**9** on NHDFs. The investigation showed that the extract and all the isolates had no toxicity to NHDFs at concentrations below 10 µM (Figure 3). Based on this, ROS screening was performed at concentrations of ≤10 μM.

### 2.3. The Effect of Hot Water Extracts of R. rugosa Flower Buds and Compounds ***1**–**9*** on TNF-α-Induced Intracellular ROS Production in NHDFs 

**Figure 4:**
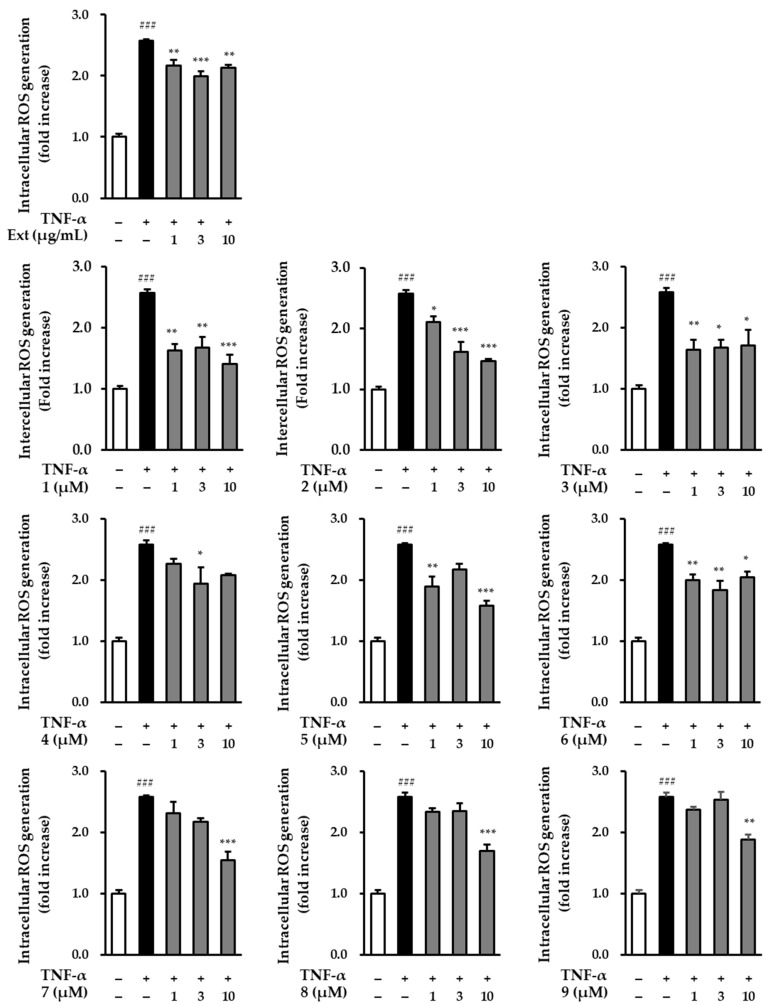
Intracellular ROS production of hot water extracts of *R. rugosa* and compounds **1**–**9** in TNF-α-induced NHDFs. The cells were treated with 1–10 (μg/mL and μM) concentrations of compounds for 1 h, and treated with TNF-α at 20 ng/mL for 15 min. The effects of these compounds were investigated using the DCFDA assay. The data are presented as the mean ± S.E.M (*n* = 3). ^###^ *p* < 0.001 control group vs. TNF-α-induced group. * *p* < 0.05, ** *p* < 0.01, and *** *p* < 0.001 sample treatment group versus TNF-α-induced group.

UV activates TNF-α receptors on the surface of skin cells, and increased TNF-α levels induce intracellular ROS production [38]. Therefore, we screened the effects of the *R. rugosa* extracts and compounds **1**–**9** on intracellular TNF-α-induced ROS production in NHDFs. In comparison to the control group, ROS generation in the group treated with TNF-α showed a significant increase (2.57 ± 0.02-fold; *p* < 0.001). Conversely, in the group treated with *R. rugosa* extracts, the level of TNF-α-induced ROS production was found to be reduced. Similarly, most of the nine compounds also suppressed the level of ROS at a concentration of 10 μM (Figure 4) [2.13 ± 0.04-fold (*p* < 0.01; 10 μg/mL of extract), 1.41 ± 0.15-fold (*p* < 0.001; 10 μM of **1**), 1.46 ± 0.04-fold (*p* < 0.001; 10 μM of **2**), 1.71 ± 0.25-fold (*p* < 0.05; 10 μM of **3**), 2.08 ± 0.02-fold (10 μM of **4**), 1.58 ± 0.07-fold (*p* < 0.001; 10 μM of **5**), 2.04 ± 0.10-fold (*p* < 0.05; 10 μM of **6**), 1.54 ± 0.14-fold (*p* < 0.001; 10 μM of **7**), 1.69 ± 0.11-fold (*p* < 0.001; 10 μM of **8**), and 1.87 ± 0.09-fold (*p* < 0.01; 10 μM of **9**)].

### 2.4. The Effects of Rosarugosides D (***1***) and A (***2***) on TNF-α-Induced Intracellular ROS Production in NHDFs

**Figure 5:**
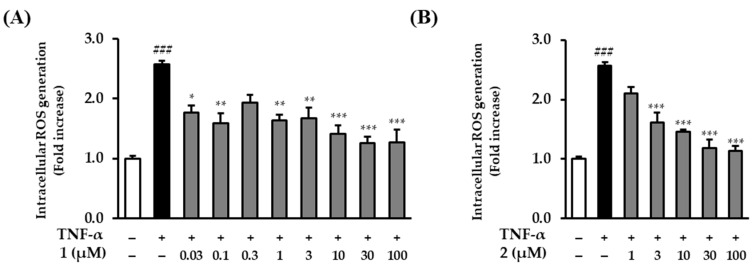
Intracellular ROS production by rosarugosides D (**1**) and A (**2**) in TNF-α-induced NHDFs. (**A**) The cells were treated with 0.03–100 μM concentrations of rosarugoside A (**2**) for 1 h, and treated with TNF-α at 20 ng/mL for 15 min. (**B**) The cells were treated with 1–100 μM concentrations of rosarugoside D (**1**) for 1 h, and treated with TNF-α at 20 ng/mL for 15 min. The effects of these compounds were investigated using the DCFDA assay. The data are depicted as mean ± S.E.M (*n* = 3). ^###^ *p* < 0.001 control group versus TNF-α-induced group. * *p* < 0.05, ** *p* < 0.01, and *** *p* < 0.001 sample treatment group versus TNF-α-induced group.

To compare rosarugoside D (**1**), which significantly inhibited ROS generation in a previous screening, and rosarugoside A (**2**), which was similar in structure to compound **1**, an experiment was conducted by adding additional concentrations. Before comparing ROS generation, the cytotoxicity of the added concentrations of compounds **1** and **2** was also investigated. Neither compound was toxic to NHDFs at concentrations below 100 µM (Figure S10). Rosarugoside D (**1**) significantly reduced the levels of ROS production at concentrations of 0.03–100 µM. At higher concentrations, such as 10, 30, and 100 µM, it was reduced 1.41 ± 0.15-fold (*p* < 0.001 at 10 µM), 1.26 ± 0.10-fold (*p* < 0.001 at 30 µM), and 1.26 ± 0.21-fold (*p* < 0.001 at 100 µM). Additionally, even at low concentrations of 0.03 and 0.1 µM, it was reduced 1.77 ± 0.12-fold (*p* < 0.01 at 0.03 µM) and 1.60 ± 0.16-fold (*p* < 0.01 at 0.1 µM) (Figure 5A). Rosarugoside A (**2**) decreased concentration-dependently at concentrations of 1–100 µM (1.62 ± 0.17-fold (*p* < 0.001 at 3 µM), 1.46 ± 0.04-fold (*p* < 0.001 at 10 µM), 1.19 ± 0.15-fold (*p* < 0.001 at 30 µM), and 1.14 ± 0.08-fold (*p* < 0.001 at 100 µM)) (Figure 5B).

### 2.5. The Effects of Rosarugosides D (***1***) and A (***2***) on TNF-α-Induced MMP-1 and Procollagen Type Ι α1 Expressions in NHDFs

**Figure 6:**
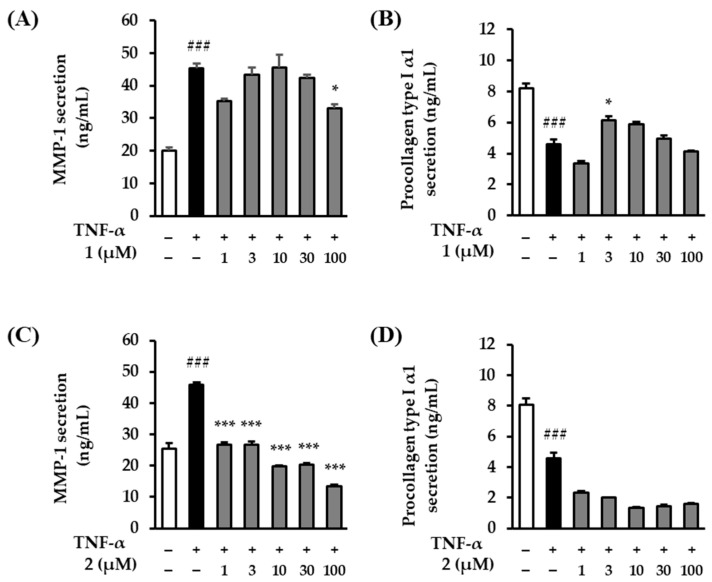
MMP-1 and procollagen type Ι α1 production of rosarugosides D (**1**) and A (**2**) in TNF-α-induced NHDFs. (**A**,**B**) The cells were pre-treated with 1–100 μM concentrations of rosarugoside D (**1**) for 1h, and treated with TNF-α at 20 ng/mL for 24 h. (**C**,**D**) The cells were treated with 1–100 μM concentrations of compound rosarugoside A (**2**) for 1 h, and treated with TNF-α at 20 ng/mL for 24 h. The effects of the compounds were investigated using an ELISA kit. The data are depicted as mean ± S.E.M (*n* = 3). ^###^*p* < 0.001 control group versus TNF-α-induced group. *p < 0.05, and ***p < 0.001 sample treatment group versus TNF-α-induced group.

TNF-α decomposes collagen by activating ROS and MMP-1 [39]. Therefore, we evaluated the effects of rosarugosides D (**1**) and A (**2**) on TNF-α-induced MMP-1 and procollagen type Ι α1 expressions in NHDFs. The two treatment groups of compounds **1** and **2** showed slightly different tendencies for the levels of MMP-1 and procollagen type Ι α1 expression. The expression of the TNF-α treatment group significantly increased compared to the control group, rising by 45.36 ± 1.40-ng/mL (*p* < 0.001). Regarding the effect of rosarugoside D (**1**) on MMP-1 secretion, MMP-1 secretion decreased by 32.96 ± 1.20-ng/mL (*p* < 0.05) at 100 μM (Figure 6A). In the case of procollagen type Ι α1, the expression of the TNF-α treatment group significantly decreased compared to the control group, falling by 4.9 ± 0.33-ng/mL (*p* < 0.001). In the rosarugoside D (**1**) treatment group, there was a tendency to recover at 3 and 10 μM (6.14 ± 0.28-ng/mL (*p* < 0.05 at 3 μM), 5.89 ± 0.15-ng/mL (at 10 μM)) (Figure 6B). As a result of rosarugoside A (**2**) addition, in the case of MMP-1, the TNF-α treatment group significantly increased compared to the control group, rising by 45.76 ± 0.80-ng/mL (*p* < 0.001). In contrast to rosarugoside D (**1**), in the rosarugoside A (**2**) treatment group, MMP-1 significantly decreased at all concentrations (26.66 ± 0.90-ng/mL (*p* < 0.001 at 1 μM), 26.76 ± 1.00-ng/mL (*p* < 0.001 at 3 μM), 19.86 ± 0.10-ng/mL (*p* < 0.001 at 10 μM), 20.46 ± 0.50-ng/mL (*p* < 0.001 at 30 μM), and 13.56 ± 0.40-ng/mL (*p* < 0.001 at 100 μM)) (Figure 6C). In the case of procollagen type Ι α1, the expression of the TNF-α treatment group significantly decreased compared to the control group, falling by 4.59 ± 0.33-ng/mL (*p* < 0.001). In contrast to rosarugoside D (**1**), the expression of procollagen type Ι α1 did not recover at any concentration in the rosarugoside A (**2**) treatment group (Figure 6D).

## 3. Discussion

The skin is mostly affected by external environmental factors, and exposure to ultraviolet rays, among other factors, can damage the skin [40]. The skin is largely composed of the epidermis and dermis. The epidermis is the outermost layer of the skin and contains melanocytes, which primarily determine skin color [41]. Additionally, the epidermis protects the body from the external environment and supplies nutrition to the skin through substance exchange [42,43]. The dermis is located below the epidermis, and several functional elements, such as blood vessels, nerves, and sweat glands, are located in the dermis [44]. In addition, proteins such as collagen and elastic fibers are abundantly distributed, maintaining the skin’s elasticity and providing structural support [45].

Exposure to ultraviolet rays is necessary to induce the production of vitamin D, but long-term exposure to ultraviolet rays damages the dermis and fibroblasts and reduces the collagen content [46,47]. Additionally, ultraviolet rays induce pro-inflammatory cytokines such as TNF-α [48]. TNF-α causes inflammatory reactions in the skin and the excessive production of intracellular ROS [38,49]. This ROS production induces the expression of MMPs. When collagen is broken down by MMPs, the ECM that maintains the elasticity of the skin and its structure collapses, causing sagging, wrinkles, and the loss of skin elasticity [50]. In addition, ROS are associated with various skin diseases such as eczema, contact dermatitis, melanoma, and skin cancer; therefore, suppressing TNF-α-induced ROS secretion is an important factor in preventing skin aging and disease [21,51,52,53].

Today, consumers are becoming more interested in health and skin aging, and the anti-aging cosmetics industry is attracting attention. Natural products are attracting attention in research on traditional and alternative medicine [54]. Ascorbic acid, polyphenols, and other natural products are antioxidants that prevent intrinsic and extrinsic aging [55]. Additionally, the awareness of the health benefits of compounds obtained from natural sources is increasing, exemplified by the development of cosmetics using natural ingredients such as green tea extract, quercetin, and resveratrol [56].

The antioxidant efficacy of *R. rugosa*, one of the natural materials, was also determined by analyzing ROS production from extracts and compounds. As a result, nine compounds taken from the hot extract of *R*. *rugosa* flowers were found to be effective in suppressing the increase in ROS production induced by TNF-α (Figure 4). Among these, rosarugoside D (**1**), a newly isolated and unreported depside, and rosarugoside A (**2**), which has a similar structure to rosarugoside D (**1**) except for the presence of an *O*-glucose at position C-8, were selected, and the production of MMP-1 and procollagen type Ι α1 was investigated.

UV and TNF-α induce various types of MMPs in the skin, such as MMP-1 (collagenase), MMP-3 (gelatinase), and MMP-9 (stromelysin-1) [57,58,59]. Among these, MMP-1 is a procollagen-degrading enzyme [60]. Procollagen type Ι α1 is the precursor molecule of type Ι collagen [61]. Collagen type Ι makes up 90% of the human body and is mainly found in skin, bones, and tendons [62]. Additionally, the induction of type Ι collagen synthesis in human fibroblasts is an important criterion for evaluating the function of cosmetic ingredients [63]. We investigated the increased levels of MMP-1 and decreased levels of procollagen type Ι α1 in rosarugosides D (**1**) and A (**2**) in TNF-α-induced NHDFs. We found that rosarugoside D (**1**) showed weak efficacy in terms of inhibiting MMP-1, but tended to restore procollagen type Ι α1. On the other hand, rosarugoside A (**2**) had a strong inhibitory effect on MMP-1 at all concentrations, but did not restore procollagen type Ι α1 (Figure 6).

These results demonstrate the potential that the pharmacological activities of *R*. *rugosa* flower buds and their depsides, rosarugosides D (**1**) and A (**2**), have in terms of preventing and protecting against TNF-α-induced skin damage. However, this study has some limitations. For example, ROS overexpression due to TNF-α activates the c-Jun/activator protein-1 (AP-1), nuclear factor kappa B (NF-κB), and transforming growth factor-beta (TGF-β) signaling pathways [64]. Through these pathways, it secretes various MMPs and induces inducible NO synthase (iNOS), cyclooxygenase-2 (COX-2), and pro-inflammatory cytokines [25,65]. However, this mechanism was not investigated in detail in this study. Additional studies are needed to understand the protective effects of rosarugosides D (**1**) and A (**2**).

## 4. Materials and Methods

### 4.1. Plant Material 

Dried flower buds of *R*. *rugosa* were purchased from the Kyungdong Market, Seoul, Republic of Korea, in August 2016. The plant material was authenticated by Prof. Dae Sik Jang, and a reference sample (RORU2-2016) was deposited at the College of Pharmacy, Kyung Hee University, Seoul, Republic of Korea. 

### 4.2. Extraction and Isolation

We extracted products from the dried flowers of *R*. *rugosa* (1.1 kg) by subjecting them to hot water (10 L) for 2 h twice. The crude extract (150.0 g, RORU-Ext) was homogeneously mixed with Diaion HP-20 and water. The solution was then evaporated in vacuo at 45 °C. Subsequently, the powered mixture was fractionated using open-column chromatography (CC) after packing the apparatus with the same resin, generating nine fractions (R1~R9). Subsequently, a Sephadex LH-20 CC (acetone–water = 2:8~6:4) was employed to separate fraction R5, producing eight subfractions (R5-1~R5-8). Fraction R5-3 was subjected to reverse-phase medium-pressure liquid chromatography (RP MPLC) and eluted with a gradient solvent system (C18 130 g, methanol–water = 1:9~5:5) to yield 14 subfractions (R5-3-1~R5-3-14). Compounds **3** (3.4 mg) and **4** (59.9 mg) were isolated from fraction R5-3-3 using RP MPLC (C18 26 g, methanol–water = 1:9~5:5). Fraction R5-6 was also injected into RP MPLC (C18 43 g, methanol–water = 1:9~3:7) to obtain 11 subfractions (R5-6-1~R5-6-11) and compound **5** (2.4 mg). Compound **2** (23.9 mg) was purified from fraction R5-6-7 using RP MPLC (C18 26 g, methanol–water = 1:9~3:7). Fraction R5-9 was fractionated using RP MPLC (C18 130 g, methanol–water = 1:9~4:6) to produce 11 subfractions (R5-9-1~R5-9-11). For the R5-9-6, preparative HPLC equipped was performed with J’sphere ODS-M80 column (250 × 200, 4.0 μm) under the isocratic solvent system (0.5% formic acid in acetonitrile/0.5% formic acid in water = 14:86) to yield compound **1** (15.2 mg). 

Fraction R6 was chromatographed over a Sephadex LH-20 (acetone–water = 5:5) to produce 18 subfractions (R6-1~R6-18). Subfraction R6-2 was fractionated using RP MPLC (C18 130g, 0.1% formic acid in methanol/0.1% formic acid in water = 0:10~5:5) to produce nine fractions (R6-2-1~R6-2-9). Fraction R6-2-4 was further separated into five subfractions (R6-2-4-1~R6-2-4-5) using RP MPLC (C18 43 g, methanol–water = 0:10~4:6). Compound **9** (11.5 mg) was isolated from R6-2-4-1 via preparative HPLC [Gemini NX-C18 110A column (250 × 21.2 mm i.d., 5 μm), 0.1% formic acid in acetonitrile/0.1% formic acid in water = 0:10~4:6]. Compound **8** (6.9 mg) was obtained via recrystallization with cold methanol from fraction R6-2-4-2. For the fraction R6-7, an RP MPLC was conducted. We performed elution with a gradient solvent system (C18 130 g, methanol:water = 3:7~6:4) to yield compounds **6** (9.5 mg) and **7** (17.0 mg).

Rosarugoside D (**1**): dark red powder; [α]_D_^21^: −36.9° (*c* 0.1, MeOH); UV λ_max_ (logε) 210 (4.02), 224 (4.01), and 271 (4.15) nm; FT-IR (ATR) ν_max_ 1357, 1513, 1607, 1668, 1750, 2886, 2968, 3332, 3679, 3761 cm^−1^; HR-MS (positive mode) *m*/*z* 505.09658 [M+Na]^+^ (calculated for C_21_H_22_NaO_13_, 505.09581); and ^1^H- and ^13^C-NMR data. This is shown in Table 1.

### 4.3. Identification of Sugar

To conduct absolute structural elucidation of sugars, acid hydrolysis was performed on compound **1** (1.0 mg) by dissolving it in 2 M trifluoroacetic acid (TFA) and heating the solution at 60 °C for 2 h. After hydrolysis, the reaction mixtures were concentrated and then subjected to liquid–liquid extraction using water and butanol. The aqueous layer was further processed by dissolving it in a solution of 10 mg/mL l-cysteine methyl ester and pyridine, and the mixture was incubated at 60 °C for 1 h in a water bath. Then, 20.0 µL of *o*-tolyl isothiocyanate was added, and the reaction was allowed to proceed for an hour at the same temperature in order to derivatize the sugars.

Chromatographic separation of the derivatized sugars was achieved using a Vanquish UHPLC-DAD. This was equipped with Hypersil GOLD C18 column (150 × 2.1 mm, 1.9 μm, Thermo Scientific, Waltham, MA, USA) and maintained at 35 °C. The analysis was conducted over 20 min under isocratic conditions, employing a mobile phase composed of 0.5% formic acid in water and 0.5% formic acid in acetonitrile, mixed at a ratio of 80:20. The identification of d-glucose was confirmed by comparing the retention times (11.4 min) and mass spectral data obtained using an LTQ-XL ion trap mass spectrometer (Thermo Scientific) with those of authentic standards, ensuring the accurate characterization of the sugars present in the samples.

### 4.4. Cell Culture

Normal human dermal fibroblasts (NHDFs) were grown on a medium at 37 °C in a humidified atmosphere of 5% CO_2_. Dulbecco’s Modified Eagle’s medium (DMEM), supplemented with 10% fetal bovine serum (FBS) and 1% penicillin/streptomycin, was used as the culture medium.

### 4.5. Cell Viability

Cell viability was assessed in 96-well culture plates (1 × 10^4^ cells/well) using the EZ-Cytox solution. To check the toxicity of the sample, each compound was diluted in a serum-free medium to a concentration of 1–100 µM and then treated. After incubation for 24 h, the supernatant was removed and 10% EZ-Cytox in a serum-free medium was added to the samples. The mixture was then incubated at 37 °C for 1 h. Subsequently, optical density was measured using a microplate reader.

### 4.6. Intracellular Reactive Oxygen Species (ROS)

Intracellular ROS production was measured in 96-well black culture plates (1 × 10^4^ cells/well) using a 2′,7′-dichlorodihydrofluorescein diacetate (DCFDA) assay. NHDFs were exposed to the extract or compounds for 1 h and then stimulated with 20 ng/mL TNF-α and 10 μM DCFDA for 15 min at 37 °C. Subsequently, the treated NHDFs were washed with PBS after removing the supernatant. Finally, ROS production was measured using a microplate reader, assessing excitation (485 nm) and emission (530 nm) wavelengths.

### 4.7. Enzyme-Linked Immunosorbent Assay (ELISA)

ELISA was performed on 48-well culture plates (2 × 10^4^ cells/well) using MMP-1 and the procollagen type Ι α1 ELISA Kit. NHDFs were exposed to compounds for 1 h and stimulated with 20 ng/mL TNF-α for 24 h at 37 °C. After that, the experiment was performed using the supernatant according to the manufacturer’s instructions. Optical density was measured using a microplate reader at 450 nm.

### 4.8. Statistical Analysis

The data are presented as the mean ± standard deviations (SD) and as the standard error of the mean (S.E.M). Differences between treatment groups were analyzed using one-way analysis of variance, and significance was defined by Tukey’s test as a level of *p* < 0.05. The data were analyzed using GraphPad Prism version 8.0.1 (GraphPad Software Inc., La Jolla, CA, USA).

## 5. Conclusions

In conclusion, the anti-skin-aging effects of *R. rugosa* flowers and their phenolic compounds were successfully validated, and our results were in agreement with previous investigations. As a result of the phytochemical investigation, nine major compounds (**1**–**9**), including one newly reported depside (rosarugoside D (**1**)), were isolated using repetitive chromatography. Among the isolated compounds, the principal depsides, which is to say rosarugosides D (**1**) and A (**2**), were found to exert antioxidant effects by inhibiting TNF-α-induced ROS overexpression in NHDFs. In addition, rosarugoside A (**2**) significantly inhibited the secretion of MMP-1, a collagen-decomposing enzyme, whereas rosarugoside D (**1**) increased the expression of procollagen type Ι α1, a collagen precursor. These results suggest that rosarugosides D (**1**) and A (**2**) can act as potential protective agents against TNF-α-induced skin damage. Additionally, rosarugosides D (**1**) and A (**2**) require additional research in order to investigate how skin damage, immunity, and inflammatory responses occur in skin cells, such as keratinocytes and dermal fibroblasts. These questions should be explored through studies into pro-inflammatory cytokine expression and the mechanisms behind it.

## Figures and Tables

**Table 1:** ^1^H- and ^13^C-NMR spectroscopic data of compound **1** (0.1% C_2_DF_3_O_2_ (TFA) in D_2_O, 500 and 125 MHz, ^a^ overlapped signal).

Position	*δ*_H_ Multi (*J* in Hz)	*δ*_C_	Position	*δ*_H_ Multi (*J* in Hz)	*δ*_C_
1		109.4	4′		150.5
2		156.4	5′	6.94 d (8.5)	115.7
3	6.67 d (2.5)	101.5	6′	7.59 dd (8.5, 2.0)	124.4
4		156.4	7′		166.4
5	6.48 d (2.0)	104.6	Glc-1″	5.04 d (7.0)	100.9
6		150.2	Glc-2″	3.55–3.61 m ^a^	72.8
7	3.61 m ^a^	29.0	Glc-3″	3.55–3.61 m ^a^	75.6
8		176.0	Glc-4″	3.48 t (9.5)	69.3
1′		119.9	Glc-5″	3.55–3.61 m ^a^	76.2
2′	7.55 d (2.5)	117.3	Glc-6″	3.74 dd (12.5, 5.5) 3.92 dd (12.5, 2.0)	60.5
3′		143.9			

## Data Availability

Data are contained within the article and Supplementary Materials.

## Supplementary Materials

The following supporting information can be downloaded at: https://www.mdpi.com/article/10.3390/plants13091266/s1, Instruments and reagents; Figure S1. HR-MS spectrum of compound **1**; Figure S2. ^1^H-NMR spectrum of compound **1** [500 MHz, 0.1% C_2_DF_3_O_2_ (TFA) in D_2_O]; Figure S3. ^13^C-NMR spectrum of compound **1** [125 MHz, 0.1% C_2_DF_3_O_2_ (TFA) in D_2_O]; Figure S4. ^1^H-^13^C HSQC spectrum of compound **1**; Figure S5. ^1^H-^1^H COSY spectrum of compound **1**; Figure S6. ^1^H-^13^C HMBC spectrum of compound **1**; Figure S7. ^1^H-^1^H NOESY spectrum of compound **1**; Figure S8. IR spectrum of compound **1**; Figure S9. Extracted Ion Chromatogram (EIC) for sugar analysis of L-glucose, D-glucose and, hydrolyzed compound **1**; Figure S10. The effect of compounds **1** and **2** on NHDF cell viability. The cells were treated with (1–100 µM) concentrations of the compound for 24 h. The effects of the compounds on cell viability were evaluated using EZ-Cytox solution. The data are presented as the mean ± SD (*n* = 3).
