# Supplementary material for: Rosarugosides A and D from Rosa rugosa Flower Buds: Their Potential Anti-Skin-Aging Effects in TNF-α-Induced Human Dermal Fibroblasts
Source: Plants (Basel). 2024 May 2;13(9):1266. doi: 10.3390/plants13091266 (PMC11085274; doi:10.3390/plants13091266)

plants

Supplementary Materials

Rosarugosides A and D from *Rosa rugosa* Flower Buds: Their Potential Anti-Skin-Aging Effects in TNF-α-Induced Human Dermal Fibroblasts

Kang Sub Kim [1], So-Ri Son [2], Yea Jung Choi [1], Yejin Kim [2], Si-Young Ahn [3], Dae Sik Jang [2,*] and Sullim Lee [3,*]

[1] College of Korean Medicine, Gachon University, Seongnam 13120, Republic of Korea; kangsub@gachon.ac.kr (K.S.K.); domdada22@gachon.ac.kr (Y.J.C.)
[2] Department of Biomedical and Pharmaceutical Sciences, Graduate School, Kyung Hee University, Seoul 02447, Republic of Korea; allosori@khu.ac.kr (S.-R.S.); yezeen@khu.ac.kr (Y.K.)
[3] Department of Life Science, College of Bio-Nano Technology, Gachon University, Seongnam 13120, Republic of Korea; sy990303@gachon.ac.kr
* Correspondence: author: dsjang@khu.ac.kr (D.S.J.); sullimlee@gachon.ac.kr (S.L.)

Contents

Instruments and reagents

Supplementary figures

Figure S1. HR-MS spectrum and the element analysis result of compound **1**.

Figure S2. ^{1}H-NMR spectrum of compound **1** [500 MHz, 0.1% $C_2DF_3O_2$ (TFA) in D_2O].

Figure S3. ^{13}C-NMR spectrum of compound **1** [125 MHz, 0.1% $C_2DF_3O_2$ (TFA) in D_2O].

Figure S4. ^{1}H-^{13}C HSQC spectrum of compound **1**.

Figure S5. ^{1}H-^{1}H COSY spectrum of compound **1**.

Figure S6. ^{1}H-^{13}C HMBC spectrum of compound **1**.

Figure S7. ^{1}H-^{1}H NOESY spectrum of compound **1**.

Figure S8. IR spectrum of compound **1**.

Figure S9. Extracted Ion Chromatogram (EIC) for sugar analysis of L-glucose, D-glucose and, hydrolyzed compound **1**.

Figure S10. The effect of compounds **1** and **2** on NHDF cell viability. The cells were treated with (1-100 μM) concentrations of the compound for 24 h. The effects of the compounds on cell viability were performed using an EZ-Cytox solution. The data were depicted as mean ± SD (*n*=3).

Instruments and reagents

For the column chromatography, Diaion HP-20 (Mitsubishi Chemical Industries, Ltd., Japan), Sephadex LH-20 (Sigma-Aldrich, USA) were used as stationary phases. Subfractions were monitored by thin-layer chromatography [silica gel 60 F254 (Merck, USA) and RP-18 F254S (Merck, USA)], together with 20% H_2SO_4 as the spray reagent. The reverse phase MPLC was applied using Combi Flash Rf200 (Teledyne Isco., USA) with Redi Sep-C18 column (26 g, 43 g, and 130 g) was used. Preparative HPLC was performed with Waters HPLC purification system [1525 pump and 996 PDA detector (Waters, USA)] equipped with preparative HPLC columns [Gemini NX-C18 110A (250 × 21.2 mm i.d., 5 µm, Phenomenex, USA) and J'sphere ODS-M80 column (250 × 200, 4.0 µm, YMC, Tokyo, Japan)].

For the structure elucidation of isolated compounds, GENESYS 10 Scanning UV/Visible Spectrophotometer (Thermo Scientific, USA) was used to measure the λ_{max} value of compounds. For the optical rotation analysis, P-2000 polarimeter (JASCO, Japan) was employed. An IR spectrum was obtained using the FT-IR-4200 (JASCO, Japan). The NMR spectrum was acquired by ECA-500MHz NMR spectrometer (JEOL, Japan). High-resolution (HR) mass spectra were collected utillizing the ESI ion source (Ionsense, Japan) coupled to an AccuTOF-TLC single-reflectron time-of-flight mass spectrometer (JEOL, Japan). For the sugar analysis, Vanquish UHPLC-DAD equipped with Hypersil GOLD C18 column (150 x 2.1 mm, 1.9 µm, Thermo scientific, USA) and LTQ-XL ion trap mass spectrometer (Thermo Scientific) were employed.

For the cell culture, normal human dermal fibroblasts (NHDF) from PromoCell GmbH (Sickingenstr, Heidelberg, Germany) were used. Dulbecco's Modified Eagle's Medium (DMEM; Gibco, Grand Island, NY, USA) and FBS (Atlas, Fort Collins, CO, USA) was utilized as the culture medium. Cell viability was assessed by MTT assay with EZ-cytox solution (DoGenBio, Seoul, Republic of Korea). A microplate reader was employed for measuring optical density values by the EnSpire multimode plate reader (PerkinElmer, Waltham, MA, USA).

To detect reactive oxygen species (ROS) production, the fluorescent probe 2',7'-dichlorodihydrofluorescein diacetate (DCFDA; Sigma-Aldrich, Burlington, USA) was utilized. The MMP-1 and procollagen type I α1 ELISA assays were conducted using ELISA Kit (R&D Systems, Inc., Minneapolis, MN, USA).

Supplementary figures

Figure S1. HR-MS spectrum and the element analysis result of compound **1**.

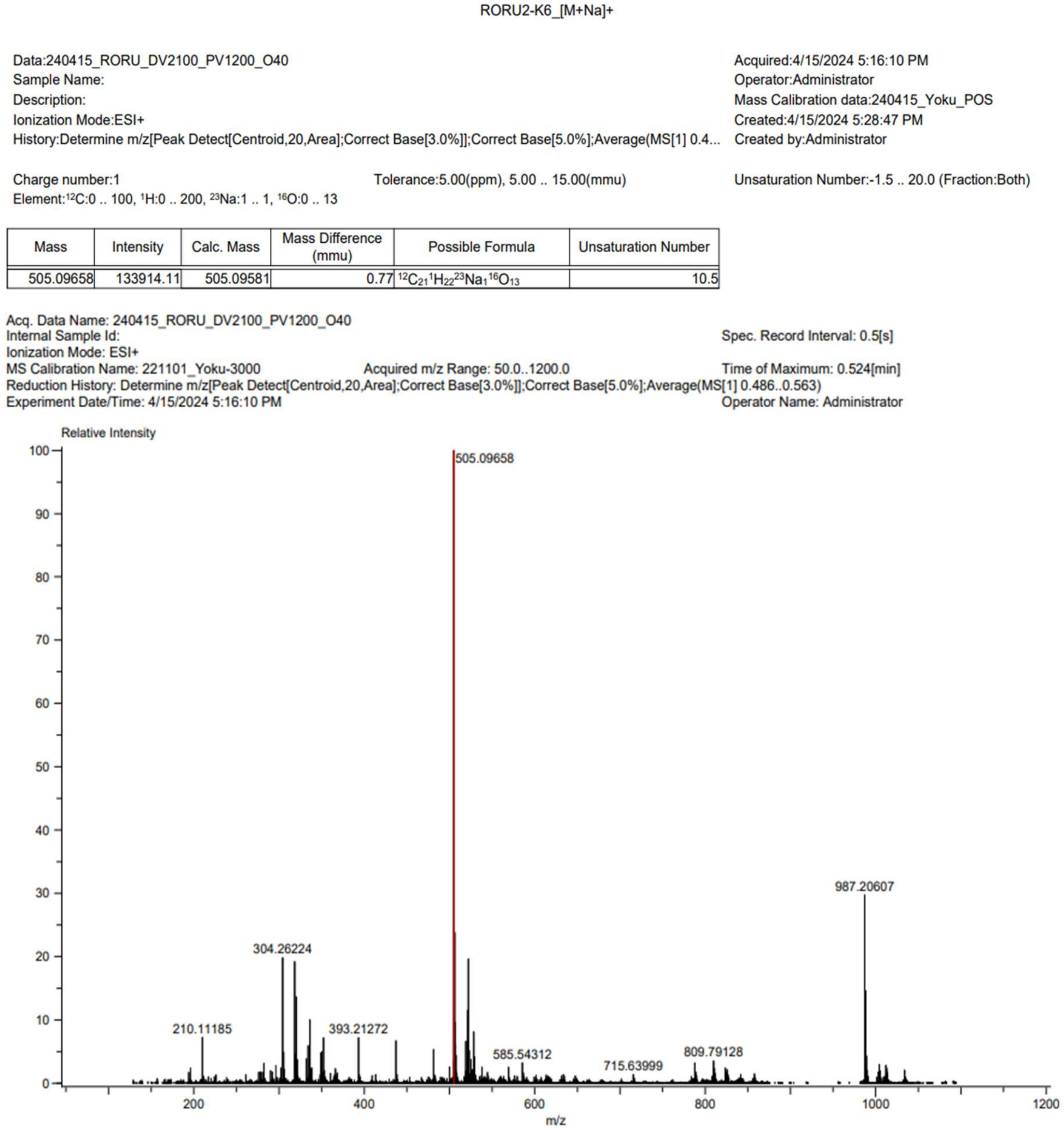

Mass	Intensity	Calc. Mass	Mass Difference (mmu)	Possible Formula	Unsaturation Number
505.09658	133914.11	505.09581	0.77	$^{12}C_{21}{}^{1}H_{22}{}^{23}Na_{1}{}^{16}O_{13}$	10.5

Figure S2. ^{1}H-NMR spectrum of compound **1** [500 MHz, 0.1% $C_2DF_3O_2$ (TFA) in D_2O].

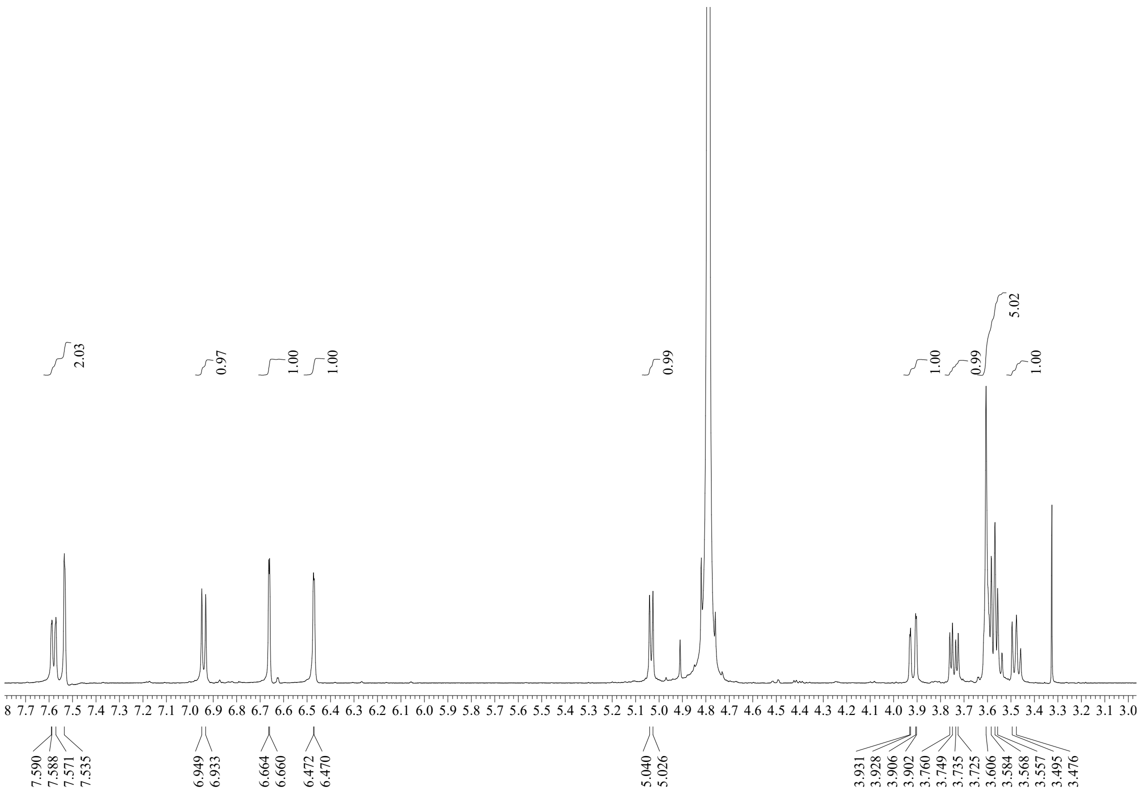

Figure S3. ^{13}C-NMR spectrum of compound **1** [125MHz, 0.1% $C_2DF_3O_2$ (TFA) in D_2O].

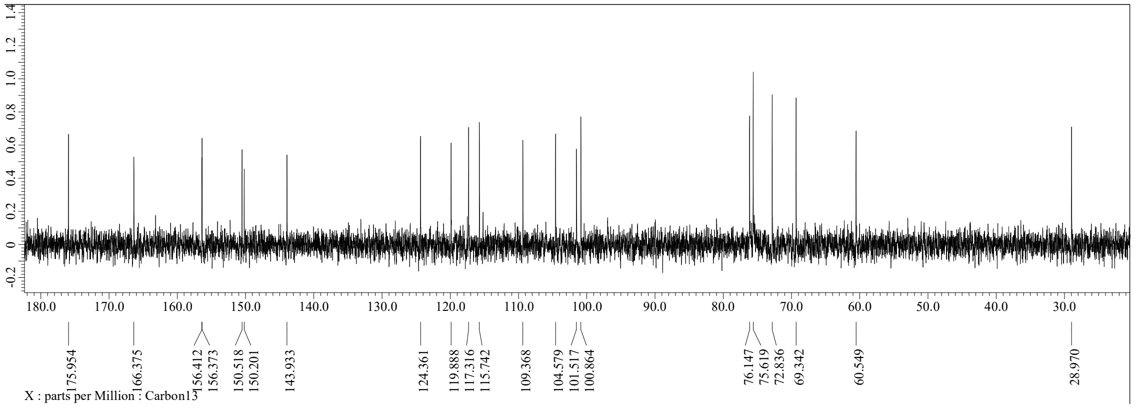

Figure S4. ^{1}H-^{13}C HSQC spectrum of compound **1**.

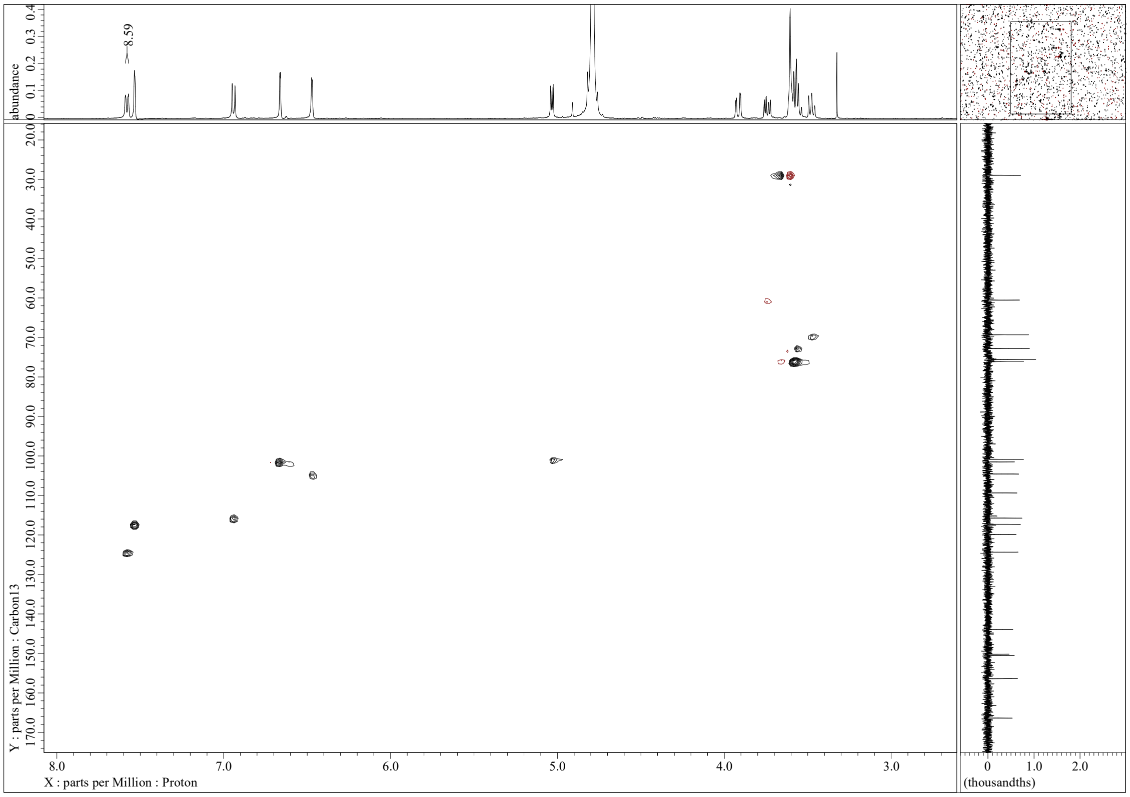

Figure S5. ^{1}H-^{1}H COSY spectrum of compound **1**.

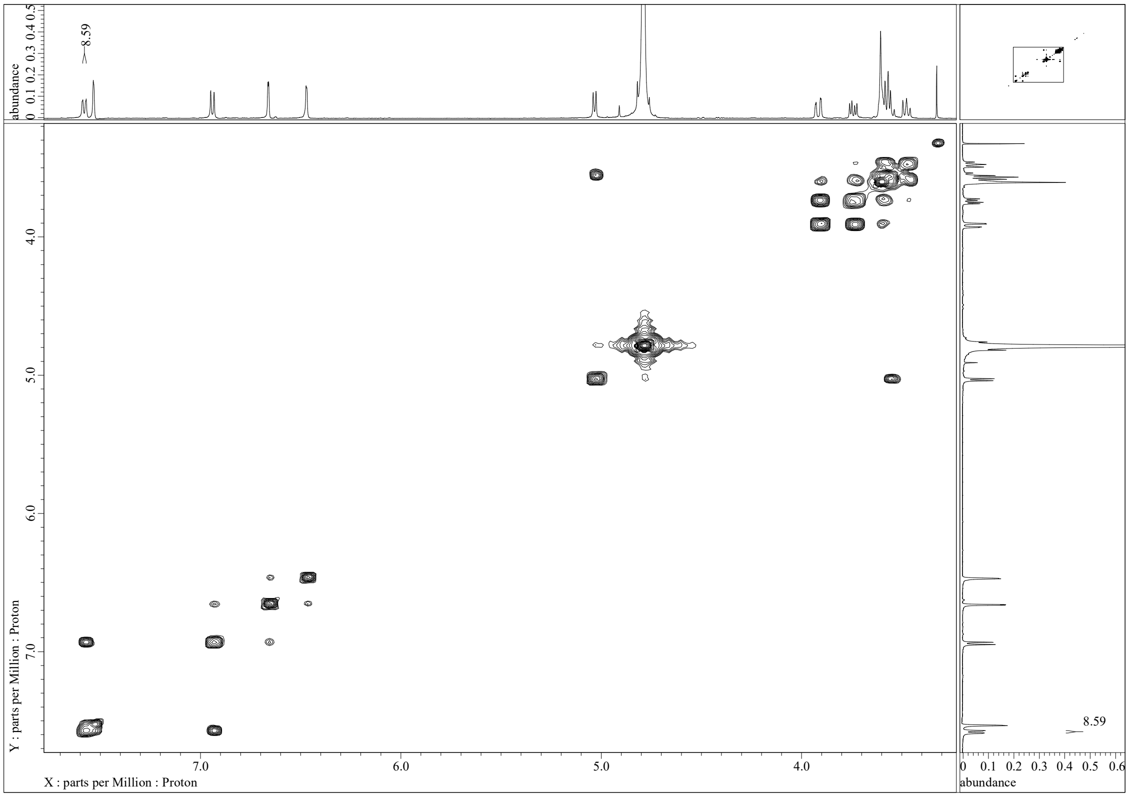

Figure S6. ^{1}H-^{13}C HMBC spectrum of compound **1**.

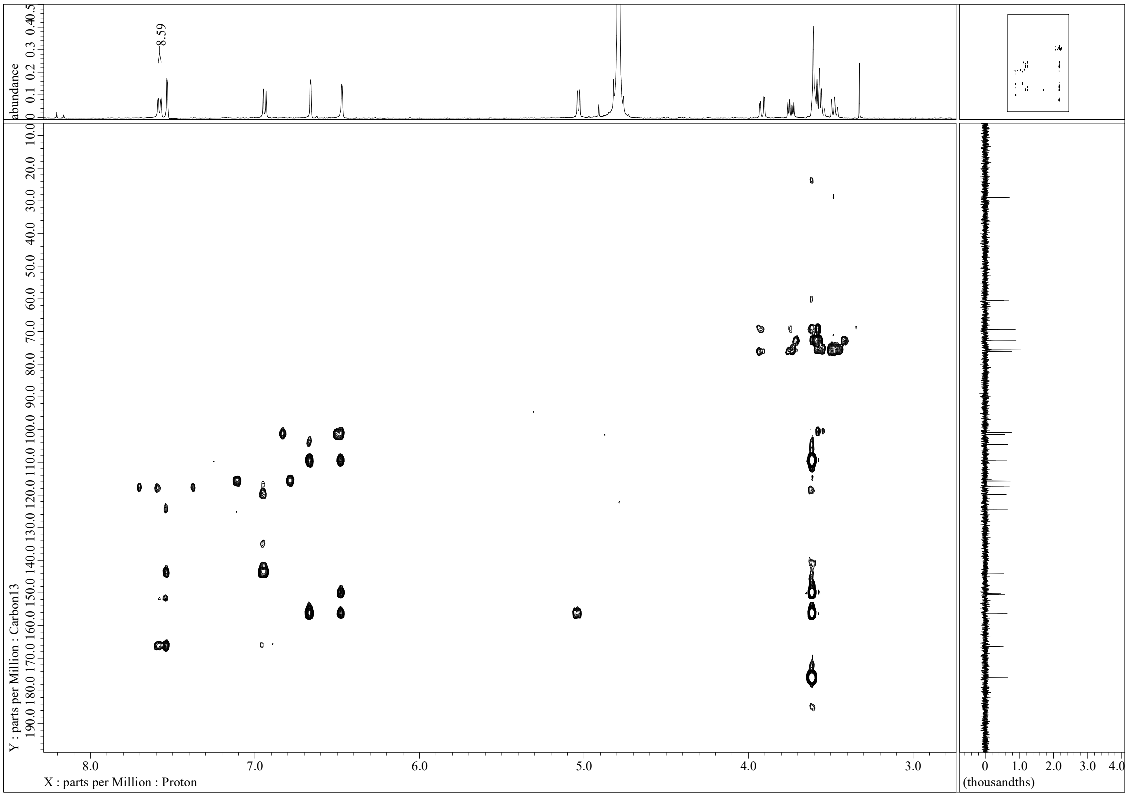

Figure S7. ^{1}H-^{1}H NOESY spectrum of compound **1**.

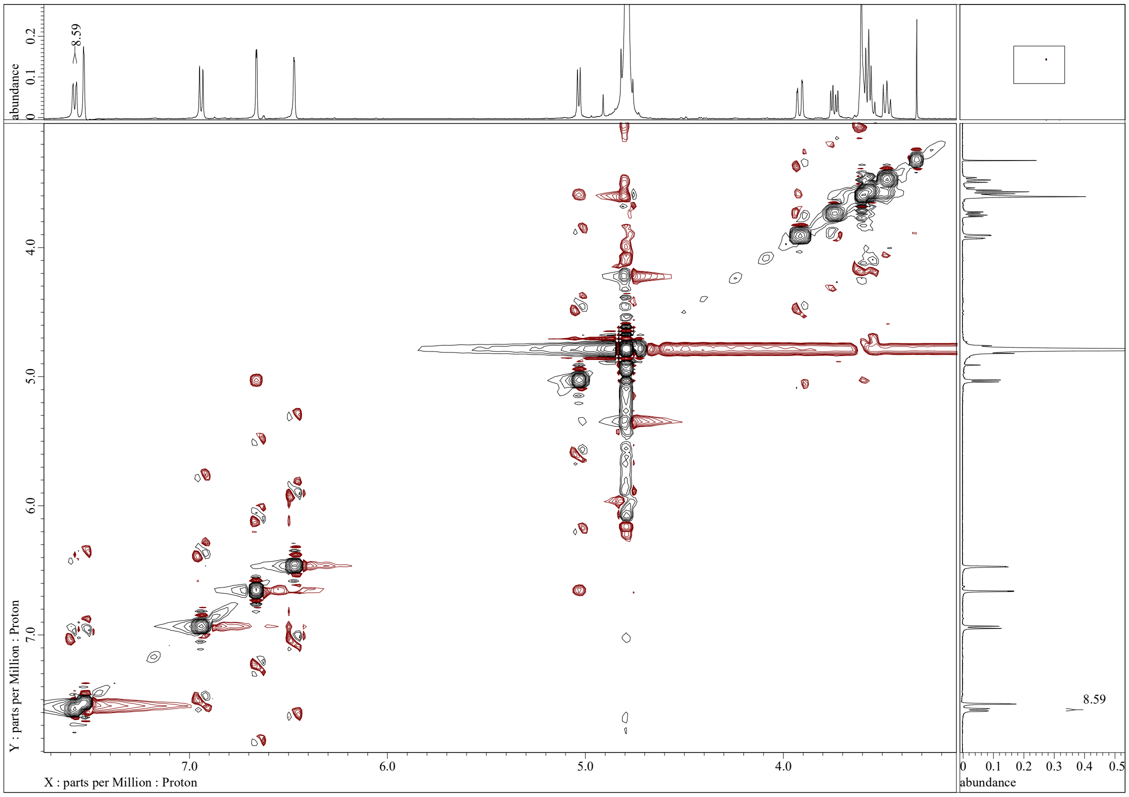

Figure S8. IR spectrum of compound **1**.

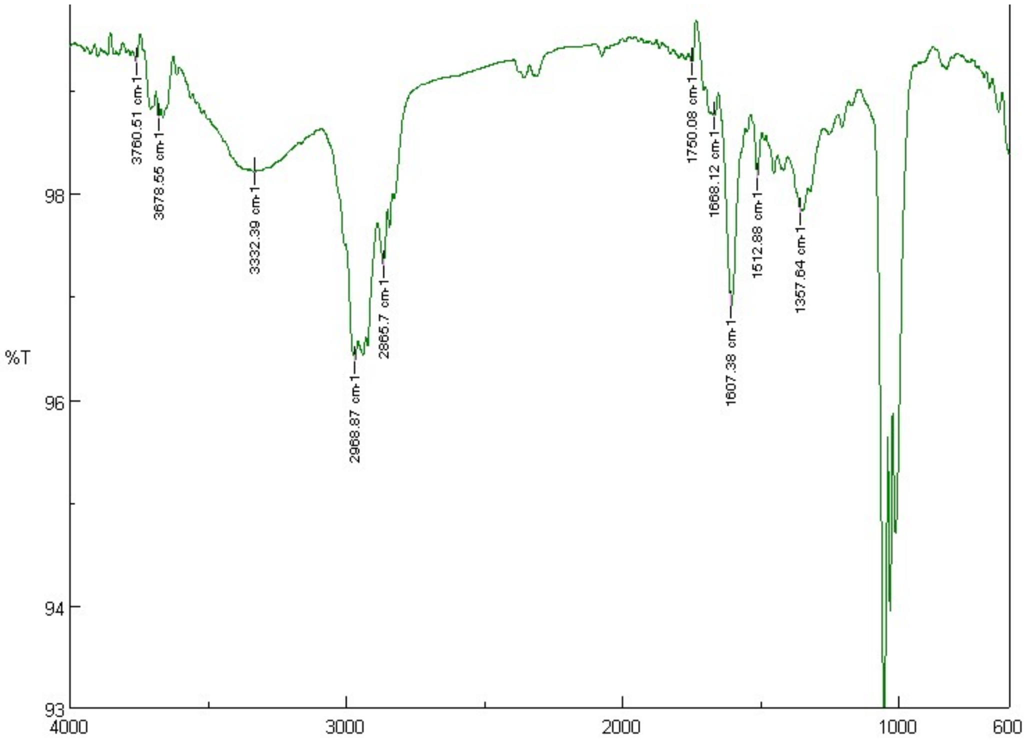

Figure S9. Extracted Ion Chromatogram (EIC) for sugar analysis of L-glucose, D-glucose and, hydrolyzed compound **1**.

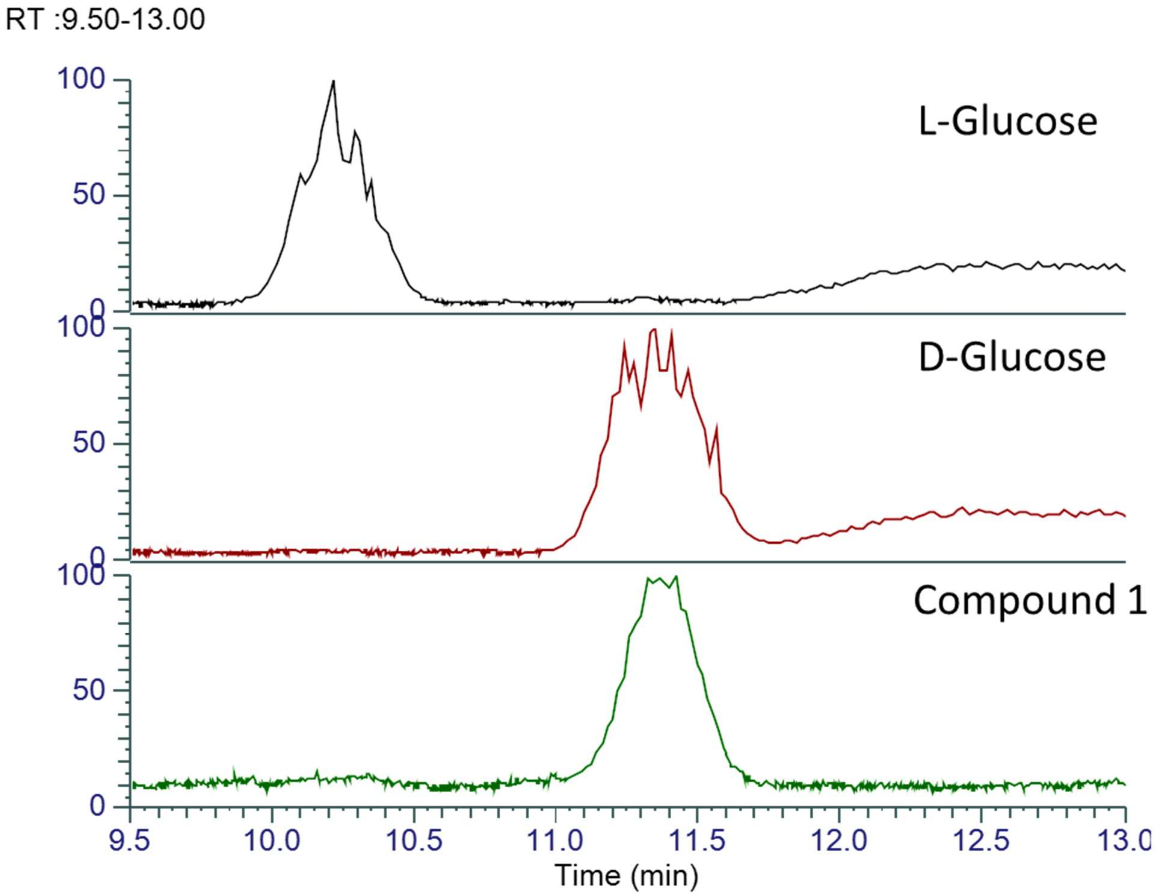

Figure S10. The effect of compounds **1** and **2** on NHDF cell viability. The cells were treated with (1-100 μM) concentrations of the compound for 24 h. The effects of the compounds on cell viability were performed using an EZ-Cytox solution. The data were depicted as mean ± SD (*n*=3).

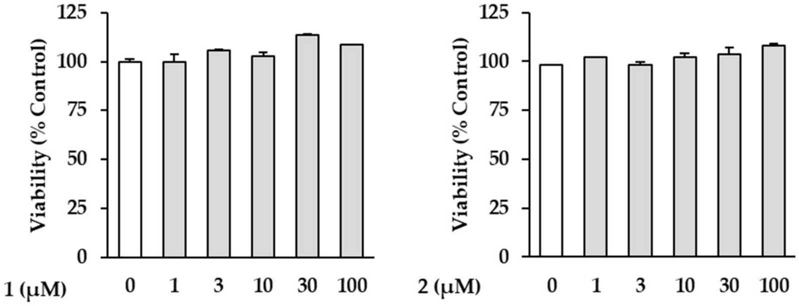

Supplement: Supplementary file 1 [file plants-13-01266-s001.zip › plants-2934245-supplementary.pdf]
